# Soil Nutrients Drive Function and Composition of *phoC*-Harboring Bacterial Community in Acidic Soils of Southern China

**DOI:** 10.3389/fmicb.2019.02654

**Published:** 2019-11-20

**Authors:** Man Man Zheng, Chao Wang, Wen Xing Li, Wen Feng Song, Ren Fang Shen

**Affiliations:** ^1^State Key Laboratory of Soil and Sustainable Agriculture, Institute of Soil Science, Chinese Academy of Sciences, Nanjing, China; ^2^University of Chinese Academy of Sciences, Beijing, China

**Keywords:** acidic soil, community structure, phosphorus deficiency, phosphate-solubilizing microorganisms, soil nutrients

## Abstract

Phosphorus (P) deficiency is an important factor that limits the agricultural production potential in acidic soils. The bacterial *phoC* gene encodes non-specific acid phosphatase (ACP), which participates in the mineralization of soil organic P and is therefore important for the improvement of soil P availability. However, the function and community population of *phoC*-harboring bacteria and their driving factors in acidic soil remain largely unknown. For this study, 51 soil samples and 207 plant samples were collected from four locations in the acidic soil region of southern China. Quantitative PCR and high-throughput sequencing were employed to analyze abundance and community composition of *phoC*-harboring bacteria. The results showed that soil P availability was the important nutrient element limiting the growth of both plants and soil bacteria. Soil ACP activity was clearly higher than alkaline phosphatase, indicating the important effect of *phoC*-harboring bacteria in acidic soils. ACP activity and *phoC* gene abundance showed a significant positive correlation, and both were closely related to soil available P, total carbon, and total nitrogen. The dominant genera of *phoC*-harboring bacteria involved *Cupriavidus*, *Stenotrophomonas*, and *Xanthomonas*. Compared to land-use pattern, sampling location, and soil parent material, soil property played a more important role in affecting *phoC*-harboring bacterial community structure, where N-related variables including soil NO${}_{3}^{-}$ -N, NH${}_{4}^{+}$ -N, and C/N ratio appeared to be the main factors. These findings suggest that *phoC*-harboring bacteria should provide an important contribution to soil P availability in acidic soil, and its function and community composition were strongly associated with soil nutrients.

## Introduction

Acidic soil (pH < 5.5) accounts for more than 50% of the potential arable land globally (Kochian et al., 2004). In China, acidic soils cover an area of 2.18 million km^2^, distributed widely in the tropical and subtropical regions of southern China (Zhao, 2002). Although these regions are rich in water and heat resources, the potential crop production is severely restricted by low pH-induced toxicity of aluminum (Al) and manganese (Mn) and nutrient deficiency of phosphorus (P), calcium (Ca), and magnesium (Mg) (von Uexküll and Mutert, 1995). Moreover, their limitations are intensified due to the accelerated rate of soil acidification (Guo et al., 2010). Among these, P deficiency is considered as the main limiting factor (von Uexküll and Mutert, 1995; Kochian et al., 2004).

In fact, most soils contain extensive stocks of total P, but the proportion of bioavailable P for plant uptake and utilization is very low (Sharma et al., 2013), which is the result of binding to soil mineral surfaces and fixation into organic forms (Kochian, 2012). In China, 42.7–47.6% of the soil is in a state of P deficiency (Tang et al., 2008), especially acidic soil, where the low level of available P makes it difficult to effectively maintain the growth of plants (Kochian et al., 2004). Furthermore, the utilization rate of P fertilizer in the current season is only 10–25%, and the residual P is quickly fixed in the soil or flow into the surface water with runoff (Kochian et al., 2004). Unfortunately, P rocks are a non-renewable resource, and P reserves are estimated to be exhausted within 50–100 years (Cordell et al., 2009). Exploiting soil-fixed P resources is an alternative approach to alleviate soil P deficiency, which not only improves plant P utilization efficiency but also reduces the requirement for chemical P fertilization (Khan et al., 2007).

Phosphate-solubilizing microorganisms (PSMs) are an important part of the ecosystem, and the main drivers for transforming soil-fixed P into bioavailable forms (Chen et al., 2006; Li et al., 2019). Regulating PSM function to improve soil P bioavailability is an effective way to alleviate soil P deficiency (Sharma et al., 2013). Previous studies mainly focused on the isolation and characterization of culturable PSM strains and their application effectiveness in soil (Kundu et al., 2009; Yu et al., 2011). For example, the strain *Pantoea dispera* was purified and its ability of soil P solubilization was tested in sterilized acidic soil (Chen et al., 2014). Collavino et al. (2010) examined a collection of microbial isolates, representing the diversity of culturable PSM from the acidic soil, and found that a number of these strains promoted plant growth.

The PSM community contains diverse microbial species, distributed throughout bacteria, fungi, and actinomycetes, and more than 20 genera have been discovered (Chen et al., 2006). The P-solubilizing biological process is mainly involved in solubilizing inorganic P and mineralizing organic P (Mohammadi, 2012). Mineralization of soil organic P in farming systems is important to improve P availability, because organic P comprises approximately 30–65% of total P in soil (Harrison, 1987). PSM possess the ability to mineralize organic P under the action of phosphatase, which hydrolyze a variety of structurally diverse phosphomonoesters to release P (Nannipieri et al., 2011). Phosphatase activity is an important indicator and reflects the mineralization potential of soil organic P. They are usually classified into acid phosphatase (ACP, EC 3.1.3.2) and alkaline phosphatase (ALP, EC 3.1.3.1) according to their optimal pH (Dick et al., 2000). Due to soil acidity, ACP should be a more predominant phosphatase form in acidic soil.

With the rapid development of molecular biology technology, numerous microbial functional genes encoding phosphatase have been discovered, and have been successfully used as marker genes to investigate uncultivated PSM species and analyze the abundance and diversity of the PSM community (Chen et al., 2017; Fraser et al., 2017; Luo et al., 2017). The genes *phoC* and *phoD* are most widely employed to study PSM communities that secrete ACP and ALP (Fraser et al., 2017), respectively. The *phoD* gene has been utilized to examine the soil PSM community, and a variety of factors including soil type, land use pattern, soil properties (e.g., soil pH, organic matter, N, and P) and fertilization are reported to affect abundance and community composition of *phoD*-harboring bacteria in various habitats (Tan et al., 2013; Fraser et al., 2015; Ragot et al., 2015, 2017). Noteworthy, Fraser et al. (2017) provided the first attempt to quantify the *phoC* gene targeting bacterial non-specific ACP in field soils. Gaiero et al. (2018) validated the *phoC* gene in a study of the microbial community, showing the genetic potential of ACP production in grassland soils. However, the understanding of *phoC*-harboring bacteria population and the primary driver of community composition in acidic soil is largely insufficient.

We hypothesized that ACP would be a dominant phosphatase form in acidic soil, and soil properties would be the primary driver of *phoC*-harboring bacterial community composition because of low pH and nutrient limitation. It is expected that, improving P-solubilizing ability of *phoC*-harboring bacterial community by regulating the driving factors will play a central role in soil P availability in acidic soil. To address our hypothesis, this study presents a field survey across 51 soil sampling sites from four locations, located in the acidic soil region of southern China. The sampling sites encompass a wide variety of soil parent material and land-use patterns. Plant and soil samples at each site were collected to characterize nutrient contents and availability and phosphatase activity. Quantitative PCR (qPCR) and high-throughput sequencing were employed to analyze soil *phoC*-harboring bacterial abundance and community structure. The aims of this study were: (1) to survey soil nutrient status in acidic soil; (2) to analyze phosphatase activity and the *phoC*-harboring bacterial community composition; (3) to illuminate which factors (e.g., soil parent material, land-use patterns, and soil property) is the dominant driver of *phoC*-harboring bacterial community structure.

## Materials and Methods

### Study Site and Sampling Description

Based on China Soil Science Database^1^, we selected four representative locations (Qiyang, Jinhua, Yingtan, and Zhaoqing) in the acidic soil region of southern China. A total of 51 soil samples and 207 plant samples were collected during May 2014. Sampling sites involved a wide variety of land-use patterns, including grasslands, woodlands, tea gardens, orchards, and farmland, as well as soil parent material, including red clay, red sandstone, and plate shale. These land-use patterns and soil parent material are the main forms in this region. The number of soil samples varied between 8 and 17 per location based on the main types of local land-use patterns and soil parent material. Plant samples originated from the most representative plants in each site. The basic sampling information, including longitude, latitude, soil parent material, land-use patterns, and plant species, are shown in Supplementary Table 1. The plant leaves were collected according to the method described by Cornelissen et al. (2003) and Bakker et al. (2011). Relatively young but fully expanded leaves (5–10 leaves) were collected at each sampling site and mixed into one plant sample. Replicate numbers per plant species are also listed in Supplementary Table 1. At each site, soil samples were taken using the grid sampling method (Fierer and Jackson, 2006; Liu et al., 2014), which established 20 m × 20 m quadrats and included five soil cores (four in each corner and one in the center). The five soil cores (0–15 cm) were collected using a 5-cm diameter auger without disturbing the plants, and mixed into one composite sample. Soil samples were thoroughly homogenized by sieving (2-mm sieve), and then immediately transported to the laboratory on ice. Each soil sample was divided into three parts. A part was stored immediately at −80°C for DNA extraction; another part was air-dried and finely ground for the determination of soil properties; and the remaining soil was stored at 4°C for assay of soil phosphatase activity, ammonium nitrogen (NH${}_{4}^{+}$ -N), and nitrate nitrogen (NO${}_{3}^{-}$ -N) within a week.

### Plant Nutrient Elements and Soil Physicochemical Property Analysis

Plant samples were dried at 70°C to constant weight, and ground (<1.0 mm) for measurement of nitrogen (N), P, and potassium (K). The ground plant samples were digested with H_2_SO_4_-H_2_O_2_. N and P concentration in the digested solution were determined according to the Kjeldahl method (Bremner, 1960) and the Bray method (Bray and Kurtz, 1945), respectively. K concentration was determined using flame photometry (FP640, Shanghai, China).

Soil pH was measured after shaking a soil water suspension (1:2.5 w/v) by a pH meter (Mettler Toledo FE20, Shanghai, China). Soil total carbon (TC) and total N (TN) were measured using a Vario MAX CNS elemental analyzer (Elementar, Hanau, Germany). Soil total P (TP) and total K (TK) were determined via digestion with H_2_SO_4_ and HClO_4_, respectively; then, TP was determined following Bray and Kurtz (1945), and TK was determined by flame photometry (FP640, Shanghai, China). Available P (AP) was extracted with a solution containing NH_4_F and HCl, and determined according to Bray and Kurtz (1945). Available K (AK) was determined in ammonium acetate extracts using flame photometry (FP640, Shanghai, China). Soil NH${}_{4}^{+}$ -N and NO${}_{3}^{-}$ -N was extracted using 2.0M KCl solution and then determined on a continuous flow analyzer (San^++^, Skalar, Holland).

### Analysis of Soil ACP and ALP Activities

The potential ACP and ALP activities were analyzed according to the protocol by Tabatabai (1994) using fresh soil. Assays were conducted in triplicate by incubating soil with *p*-nitrophenyl phosphate (Sigma-Aldrich, United States) in a modified universal buffer at pH 6.0 and 11.0, respectively. Samples were filtered and the color intensity of *p*-nitrophenol (*p*NP) was measured using a spectrophotometer at 410 nm (Tecan, Männedorf, Switzerland). The potential ACP and ALP activities were expressed as μg of *p*NP produced per g of soil (dry weight equivalent) within 1 h.

### DNA Extraction and Quantification of Gene Abundance

Soil DNA was extracted from 0.5 g of fresh soil sample with the FastDNA SPIN Kit (MP Biomedicals, Santa Ana, CA, United States) following the manufacturer’s protocol. Each soil sample contained three successive DNA extractions, and the triplicates were pooled into one DNA sample. Subsequently, the DNA was purified using a PowerClean DNA Clean-up Kit (Mobio, Carlsbad, CA, United States), following the manufacturer’s instructions. The purified DNA was quantified on a NanoDrop ND-1000 spectrophotometer (NanoDrop Technologies, Wilmington, NC, United States), and then stored at −20°C until further use.

The universal primers of target genes for bacterial 16S rRNA, fungal ITS, and *phoC*-harboring bacteria are listed in Supplementary Table 2. The gene amplification was conducted in a LightCycler 480 real-time PCR system (qPCR) (Roche Diagnostics, Mannheim, Germany). The qPCR program and reaction composition are described in Supplementary Table 2. Three replicates were analyzed for each DNA sample. To generate the standard curves, 16S rRNA, ITS, and *phoC* gene fragment was independently cloned into the pMD19-T vector (Takara Bio, Japan) and subsequently transferred into *Escherichia coli* DH5α competent cells. The plasmids containing the correct fragment length were selected, verified, and extracted. The plasmids were 10-fold serially diluted and used to construct standard curves. The PCR amplification efficiency of 16S rRNA, ITS, and *phoC* gene were 92.5% (*R*^2^ = 0.995), 107.7% (*R*^2^ = 0.995), and 98.2% (*R*^2^ = 0.993), respectively.

### High-Throughput Sequencing and Data Processing

Universal primers phoc-A-F1 and phoc-A-R1 were used for PCR amplification of *phoC* gene (Supplementary Table 2). To distinguish the sample, a sample-specific tag (7-bp barcode) was added to the forward primer. The PCR program and reaction composition are shown in Supplementary Table 2. Triplicate PCR amplifications for each sample were conducted and pooled as a PCR product. Then, the purified amplicons were pooled in equimolar volumes and subjected to paired-end sequencing using an Illumina HiSeq PE250 platform (Shanghai Personal Biotechnology, Co., Ltd.). The sequencing data has been submitted to the NCBI Sequence Read Archive (SRA) database (accession number SRP198946).

Pairs of reads from the raw data were first merged with FLASH version 1.2.7 (Magoc and Salzberg, 2011). Sequencing reads were processed with the Quantitative Insights Into Microbial Ecology (QIIME, v1.8.0) (Caporaso et al., 2010). Low-quality sequences that had a base length < 150 bp, contained ambiguous nucleotides, or did not match the primer, were removed. After the samples were sorted, barcode and primer sequences were eliminated. Subsequently, chimeric sequences (*de novo*) were examined and eliminated by using USEARCH (v5.2.236^2^). The UPARSE pipeline was used to cluster sequences into operational taxonomic units (OTUs) at 97% sequence similarity (Edgar, 2013). Representative sequences in the OTUs were taxonomically classified via BLAST algorithm-based search against NCBI’s NT (Nucleotide) database within GenBank^3^ with the following criteria required for a match: a BLAST identity > 80% and a BLAST E-value at minimum E-10. To remove heterogeneity of the number of sequences, each sample was rarefied to the identical number of reads (15,463 sequences, minimal sequencing depth) for downstream analysis.

### Statistical Analysis

The α-diversity indices, including OTU number, Chao1, Shannon, and Simpson, were generated using QIIME software. Statistical analysis was performed using the SPSS software (20.0, IBM Corporation, New York, NY, United States). Spearman’s correlation coefficients were used to test relationships between soil variables and plant elements, phosphatase activity, gene copy numbers, α-diversity indices, and relative abundances of genera. Two-tailed *t*-test was used to examine differences between ACP and ALP activity. Variation partitioning analysis (VPA) based on partial canonical correspondence analysis (pCCA) was employed to quantitatively evaluate the contribution of soil property, land use pattern, sampling site, and soil parent material to the variation of community structure (Legendre and Legendre, 2012). Canonical correspondence analysis (CCA) was further performed to explore the effect of total soil variables on community structure. Monte Carlo simulation tests were used to evaluate the relationship between each soil variable and the community composition variances (Legendre and Legendre, 2012). A multiple regression tree (MRT) was built to identify most important soil variables influencing the community structure (Matsui et al., 2018). VPA, CCA, and Monte Carlo simulation tests were conducted using the *vegan* package in R software (version 2.15.0), and MRT was performed using the *mvpart* package of R.

## Results

### Plant Leaf Nutrients, Soil Property, and Microbial Abundance

**FIGURE 1:**
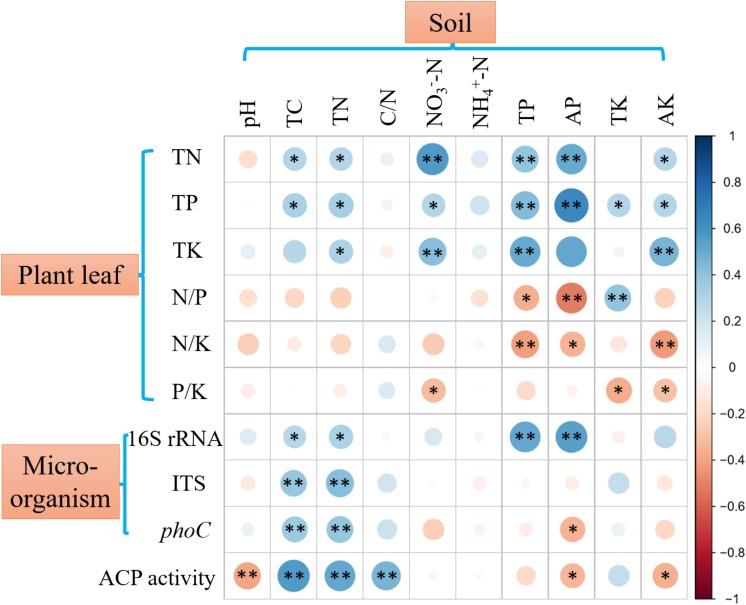
Spearman’s correlations between plant nutrients, microbial abundance, ACP activity and soil variables. 16S rRNA, 16S rRNA gene copy number; ITS, fungal ITS gene copy number; *phoC*, *phoC* gene copy number. ^∗∗^ Indicates significant correlations at *p* < 0.01; ^∗^ Indicates significant correlations at *p* < 0.05. Blue dots indicate positive correlations, and red dots indicate negative correlations.

**FIGURE 2:**
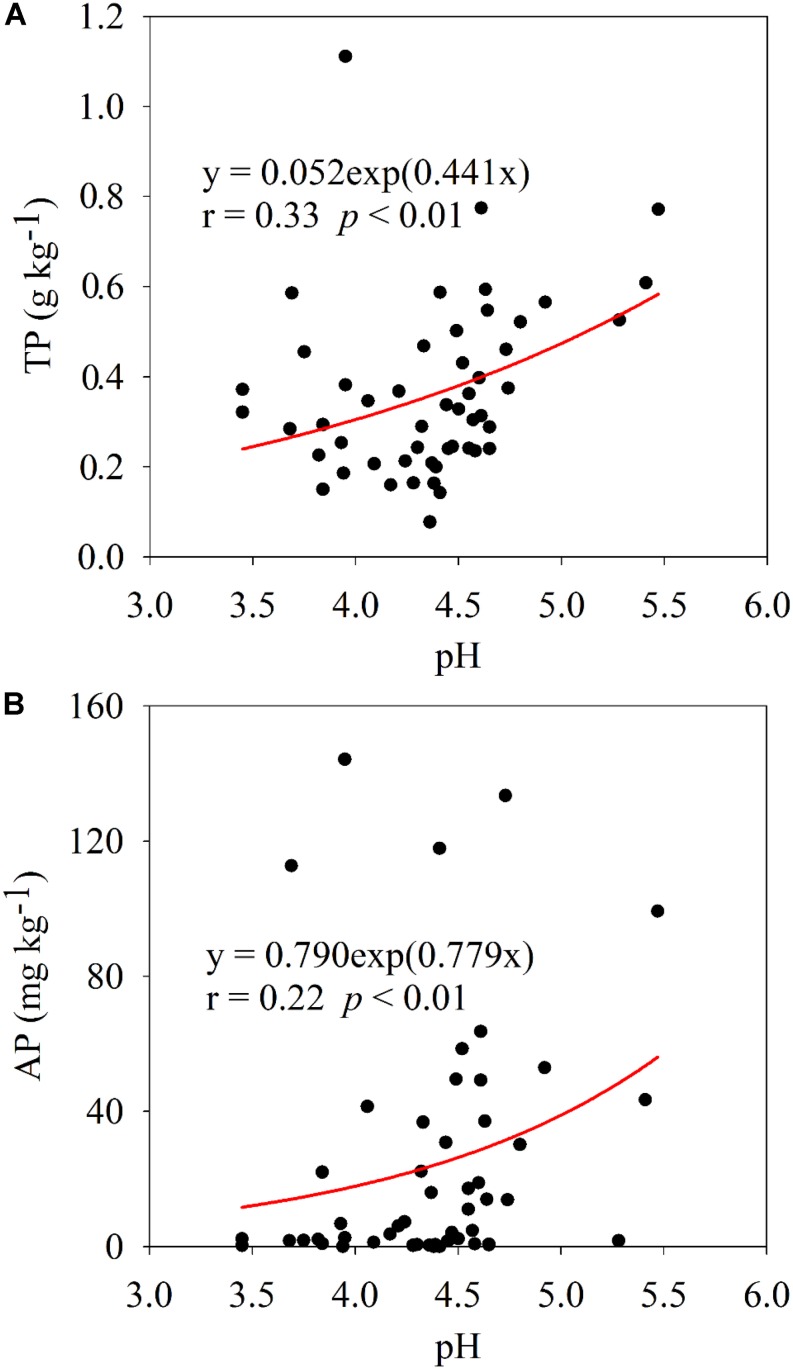
Relationship between soil pH and TP **(A)** and AP **(B)** across 51 soil samples.

Plant leaf nutrient contents and nutrient ratios are displayed in Supplementary Figure 1, and N, P, and K contents of all collected plant leaves were 2.36 ± 0.84, 0.21 ± 0.17, and 0.95 ± 0.75%, respectively. Soil property and microbial abundance are shown in Supplementary Figures 2, 3, respectively. Spearman’s correlation analysis showed that plant leaf nutrients, nutrient ratios, and soil microbial abundance had significant, but differential, correlations with soil variables (Figure 1). The contents of N, P, and K in plant leaves were positively (*p* < 0.05 or *p* < 0.01) correlated with soil TC, TN, TP, AP, NO${}_{3}^{-}$ -N, and AK, in which soil AP showed the highest correlation with plant P content. The N/P and N/K in plants were significantly (*p* < 0.01) negatively correlated with soil TP and AP. The copy number of soil bacterial 16S rRNA genes was positively (*p* < 0.01) correlated with soil TP and AP, while the copy number of soil fungal ITS genes was only correlated with soil TC and TN (*p* < 0.01). Across 51 soil samples, the average contents of soil TP and AP were 0.37 ± 0.19 g kg^–1^ and 25.31 ± 36.60 mg kg^–1^, respectively (Supplementary Figure 2), and they were significantly (*p* < 0.01) positively correlated with soil pH (Figure 2).

### Correlations Between Soil Properties and ACP Activity and *phoC* Gene Copy Number

**FIGURE 3:**
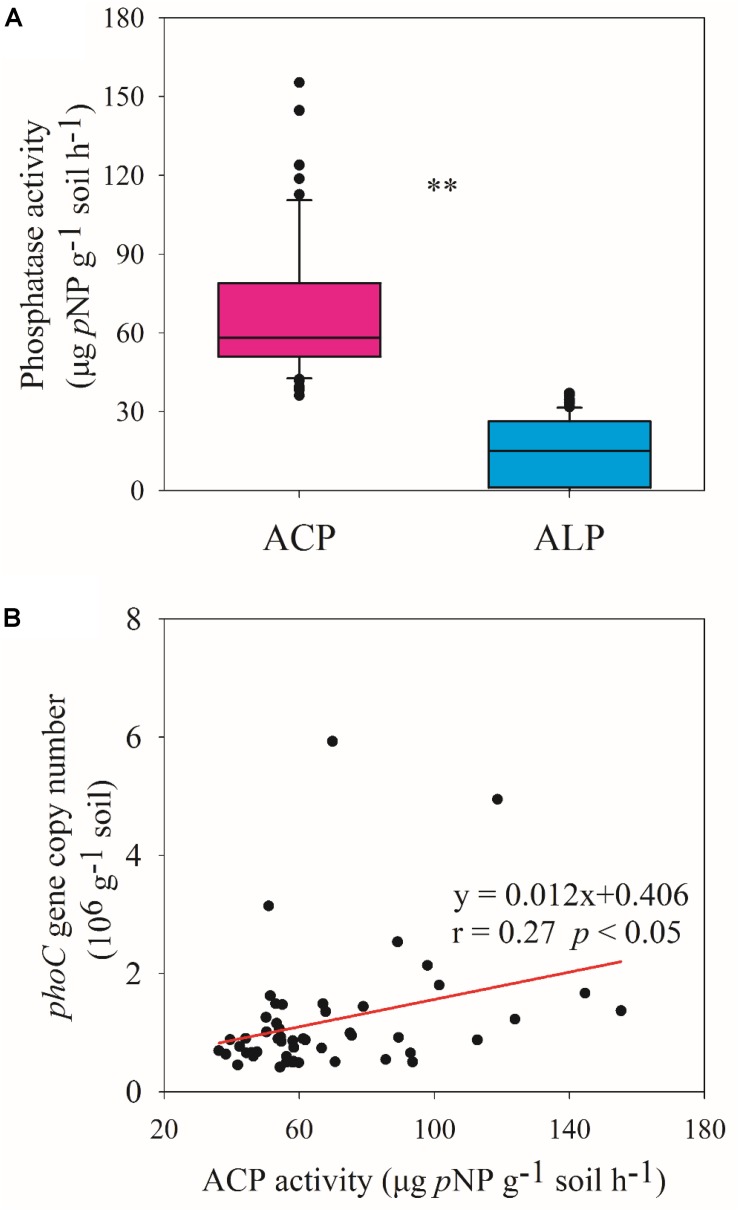
Phosphatase activity **(A)** and relationship between ACP activity and *phoC* gene copy number **(B)**. ^∗∗^ Indicates significant differences at *p* < 0.01.

The ACP activity across 51 soil samples was 67.96 ± 26.85 μg *p*NP g^–1^ soil h^–1^, which was significantly (*p* < 0.01) higher than ALP activity (15.10 ± 12.17 μg *p*NP g^–1^ soil h^–1^) (Figure 3A), showing that ACP was the more important phosphatase in acidic soil. Therefore, subsequent analyses focused on soil ACP. ACP activity and *phoC* gene copy number were found to be significantly (*p* < 0.05) positively correlated (Figure 3B), and both were negatively (*p* < 0.05) correlated with soil AP and significantly (*p* < 0.05 or *p* < 0.01) correlated with other soil variables, in which TN and TC had the highest correlation coefficient (Figure 1).

### Diversity and Composition of *phoC*-Harboring Bacterial Community

A total of 5,004,572 high-quality *phoC* gene sequences with 15,463 to 221,629 sequences per sample were obtained across 51 soil samples (Supplementary Table 3). After being rarefied to 15,463 sequences, the number of OTUs (97% similarity) per sample ranged from 27 to 153. Correlation analysis showed that OTU number, Chao1, and Shannon were positively (*p* < 0.05) correlated with C/N, and OTU number and Chao1 were also positively (*p* < 0.05) correlated with ACP activity, while Chao1 was negatively (*p* < 0.05) correlated with TP (Supplementary Figure 4).

**FIGURE 4:**
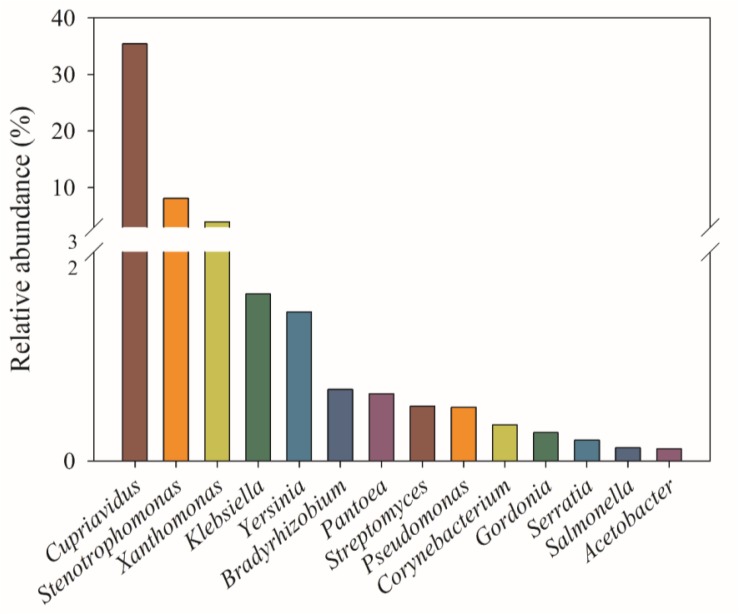
Relative abundances (%) of the 14 most abundant *phoC*-harboring bacterial genera (>0.1%) across 51 soil samples.

The genera with relative abundance above 0.1% are shown in Figure 4. The genus *Cupriavidus* was the most dominant group, accounting for 35.4% of total *phoC* gene sequences, followed by *Stenotrophomonas* (8.13%) and *Xanthomonas* (3.96%), *Klebsiella* (1.60%), *Yersinia* (1.42%), *Bradyrhizobium* (0.68%), *Pantoea* (0.64%), *Streptomyces* (0.52%), and *Pseudomonas* (0.51%). Spearman’s correlation analysis showed that soil variables were closely, but differentially, correlated with most of the abundant genera (Supplementary Figure 5). The relative abundances of *Cupriavidus*, *Klebsiella*, *Streptomyces*, and *Corynebacterium* were positively correlated with the NO${}_{3}^{-}$ -N, and *Cupriavidus* and *Klebsiella* were also positively correlated with TK and NH${}_{4}^{+}$ -N, respectively. The relative abundance of *Stenotrophomonas* and *Xanthomonas* were positively correlated with C/N, while *Stenotrophomonas* abundance was negatively correlated with TK. The *Pseudomonas* abundance was positively correlated with TC, C/N, and ACP activity. In addition, the relative abundance of *Serratia*, *Salmonella*, and *Acetobacter* were positively correlated with pH, NH${}_{4}^{+}$ -N, and TK, respectively.

### Factors Affecting *phoC*-Harboring Bacterial Community Structure

**FIGURE 5:**
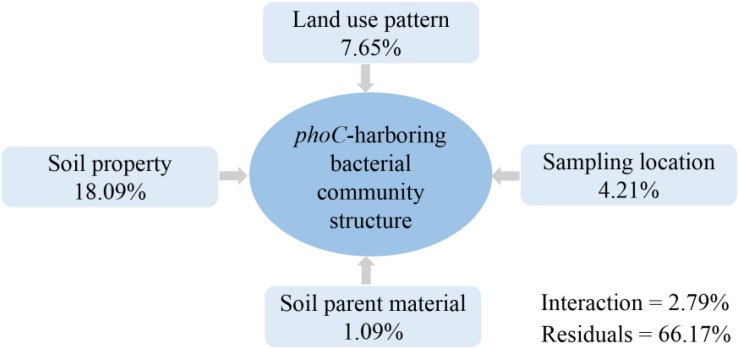
Variation partitioning analysis (VPA) of the proportion of variation in *phoC*-harboring bacterial community across 51 soil samples that can be explained by soil property, land use pattern, sampling location, soil parent material, interaction, and the residual unexplained variation.

Variation partitioning analysis showed that all evaluated factors explained a total of 33.83% of the variation of the *phoC*-harboring bacterial community structure. Compared to sampling site and soil parent material, soil property and land-use pattern provided the higher contributions, with 18.09 and 7.65%, respectively (Figure 5). This indicated soil property as the prominent driver of *phoC*-harboring bacterial community structure.

**FIGURE 6:**
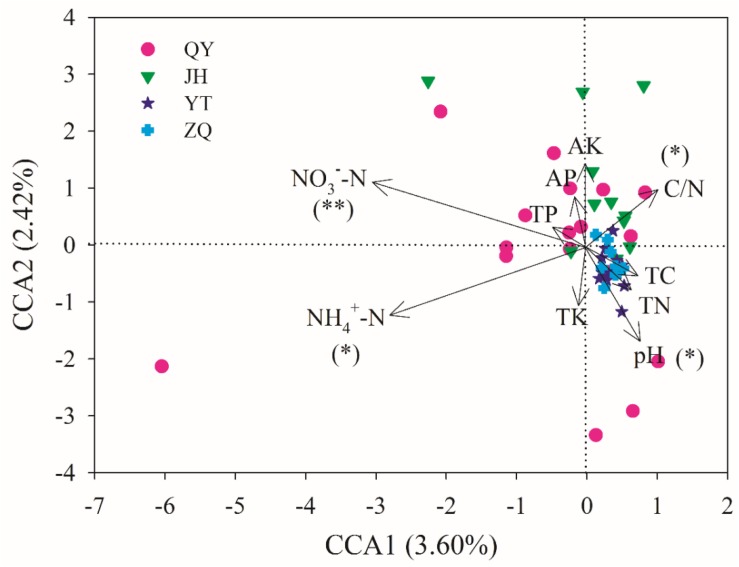
Canonical correspondence analysis (CCA) of the *phoC*-harboring bacterial community structure with soil variables across 51 soil samples. The positions and lengths of the arrows indicate the directions and strengths of the effects of soil variables, respectively. Asterisks indicate significant relationships between soil variables and community variances, tested via the Monte Carlo simulation test (^∗∗^*p* < 0.01, ^∗^*p* < 0.05).

**FIGURE 7:**
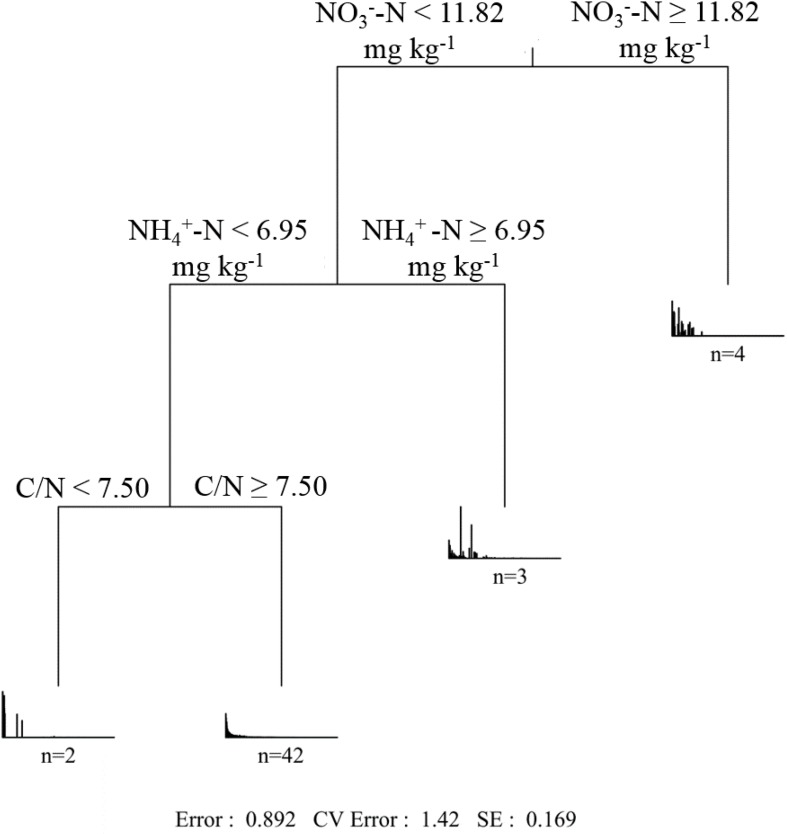
Multiple regression tree (MRT) analysis of *phoC*-harboring bacterial community and soil variables across 51 soil samples. The number of samples included in the analysis is shown below the bar plots.

The effect of soil properties on the community structure was further analyzed using the CCA (Figure 6). The first two axes explained 3.60 and 2.42% of the total variation, respectively. According to the Monte Carlo simulation test, NO${}_{3}^{-}$ -N, NH${}_{4}^{+}$ -N, pH, and C/N showed significant (*p* < 0.05 or *p* < 0.01) relationships with the community composition variances, and the influencing strength (*r*^2^ value) followed: NH${}_{4}^{+}$ -N > NO${}_{3}^{-}$ -N > pH > C/N (Supplementary Table 4). MRT analysis further indicated that the *phoC*-harboring bacterial community structure was mainly shaped by NO${}_{3}^{-}$ -N, NH${}_{4}^{+}$ -N, and C/N (Figure 7). Among these, the most important soil variable was NO${}_{3}^{-}$ -N, with a threshold of 11.82 mg kg^–1^.

## Discussion

### Soil P Status, Phosphatase Activity, and *phoC* Gene Abundance in the Acidic Soil Region of Southern China

As expected, the results of this field survey clearly showed that soil P, especially its bioavailability, was an important nutrient element affecting the plant leaf nutrient contents and soil bacteria in the acidic soil of southern China (Figure 1). The average soil AP concentration was 25.31 mg kg^–1^ across 51 samples surveyed, in which those of 27 soil samples were less than 10 mg kg^–1^ (Supplementary Figure 2). Such a low AP concentration should be difficult to maintain the normal growth of plants. Generally, plant P contents with less than 0.20% is considered as P deficient (Sharma et al., 2013); therefore, about 60% of surveyed plant samples should suffer from P deficiency (Supplementary Figure 1). Previous studies on the P deficiency in acidic soils are limited to the major crop-production areas (Kochian, 2012), fertilizer management (Zhang et al., 2009), and soil amelioration (Malik et al., 2012). The present study was conducted by actual field sampling, covering different soil parent materials, land use patterns, and plant species; therefore, it could comprehensively demonstrate the actual state of P deficiency in acidic soil ecosystem. Moreover, soil P source was positively correlated with soil pH (Figure 2), implying low pH was the vital limiting factor for P availability in acidic soil. In the surveyed area, soil acidification is accelerating due to the increased input of ammonium-based fertilizers, excessive soil utilization, and industrialization practices (Guo et al., 2010), potentially exacerbating soil P deficiency.

Phosphate-solubilizing microorganisms have been suggested as best eco-friendly means for plant P nutrition (Sharma et al., 2013), and can transform soil P into bioavailable forms by secreting phosphatase, mainly including ACP and ALP (Nannipieri et al., 2011). Previous studies have mainly focused on ALP in a range of environments (Tan et al., 2013; Ragot et al., 2017), even in the acidic soil ecosystem (Chen et al., 2017). However, this study showed that the activity of ACP was much higher than that of ALP (Figure 3A), indicating the more important role of ACP in acidic soil. Consistent with the study of Dick et al. (2000), ACP activity was higher than ALP activity across five acidic soils, where ACP activity ranged from 44 to 206 μg *p*NP g^–1^ soil h^–1^, at the same level as the present observation. Furthermore, similar to a previous study (Fraser et al., 2017), we found that ACP activity correlated positively with *phoC* gene abundance (Figure 3B), and both were clearly related to soil nutrients, especially TN, TC, and AP (Figure 1). It is not surprising that low AP conditions favor the activity of phosphatases (Nannipieri et al., 2011). When facing P scarcity, microorganisms can upregulate the expression of the specific functional genes that encode phosphatases (Vershinina and Znamenskaya, 2002). Additionally, both TN and TC as substrates contribute to the growth of various PSM species (Nahas, 2007). Luo et al. (2017) reported that organic fertilizer, which is rich in C substrates and TN but low in AP, favored the growth of *phoD*-harboring microbial species. Moreover, low pH conditions can adversely affect the function of microbes involved in C and N metabolism (He et al., 2007; Herold et al., 2012; Kunito et al., 2016), which should lead to the accumulation of soil C and N. This may also be a reason for the high activity of ACP in acidic soils.

### Dominant *phoC*-Harboring Bacterial Genera in Acidic Soils

To date, the species of PSM in acidic soils have not been studied. This study found that the dominant *phoC*-harboring bacterial genera in acidic soils were *Cupriavidus*, *Stenotrophomonas*, *Xanthomonas*, *Klebsiella*, *Yersinia*, *Bradyrhizobium*, *Pantoea*, *Streptomyces*, and *Pseudomonas* (Figure 4). Neal et al. (2018) reported that genera possessing *phoC* gene included *Caulobater*, *Stenotrophomonas*, *Methylobacterium*, *Sphingomonas*, *Xanthomonas*, and *Pseudomonas* in permanent grassland, arable, and bare fallow plots. Gaiero et al. (2018) discovered *Xanthomonadales*, *Enterobacteriales*, *Rhizobiales*, and *Burkholderiales*, as a population of *phoC*-harboring microbes in grassland soils. These results indicate that different *phoC* populations are present in different ecosystems, and imply the existence of unique *phoC*-harboring bacterial species in acidic soils. Moreover, the genus *Bradyrhizobium*, *Streptomyces*, *Actinoplanes*, *Rhodanobacter*, *Burkholderia*, *Pseudomonas*, and *Xanthomonas* often appear as the dominant genera of *phoD*-harboring microbes (Ragot et al., 2015, 2017; Chen et al., 2017; Luo et al., 2017). This clearly indicates that *Bradyrhizobium*, *Streptomyces*, *Pseudomonas*, and *Xanthomonas* exist in the form of both *phoC*- and *phoD*-harboring microbes, suggesting that the same species of PSM may secret different phosphatases (Dick et al., 2000). Similarly, the genera *Serratia*, *Pantoea*, *Pseudomonas*, and *Stenotrophomonas* have be found to solubilize inorganic P (Kundu et al., 2009; Yu et al., 2011); thus, these PSM species may be involved in both mineralization of organic P and solubilization of inorganic P.

In the surveyed acidic soils, *Cupriavidus* was the dominant *phoC*-harboring bacterial genus. In addition to the mineralization of organic P (Qian et al., 2010), the genus *Cupriavidus* was reportedly able to resist metal toxicity by expressing relevant resistance genes (Janssen et al., 2010), which may help adapt the acidic soil with high Al toxicity. Moreover, a P-solubilizing isolate of *Cupriavidus* from a contaminated soil showed a high acid and heavy metal tolerance (Kuppusamy et al., 2016). Thus, *Cupriavidus* species as good candidates could be applied in P-mineralization of acidic soil. Other *phoC*-harboring species, including *Klebsiella*, *Stenotrophomonas*, and *Serratia* could alleviate metal ion damage by secreting organic acids or producing siderophores (Mora et al., 2017); however, *Pseudomonas fluorescens* exhibits a fine metabolic balancing act to adapt Al toxicity (Lemire et al., 2010). These diverse mechanisms equip them with a better ability to adapt to an acidic soil environment.

### Drivers of the *phoC*-Harboring Bacterial Community Structure

Various factors, such as soil properties, soil parent material, management, and crop type, have been reported to affect the composition of the PSM community (Ragot et al., 2017; Neal et al., 2018). In the present study, most of these factors affected the *phoC*-harboring bacterial community structure, in which soil property was the prominent driver (Figure 5). Similarly, the *phoD*-harboring bacterial community correlated significantly with soil property in several studies (Chen et al., 2017; Luo et al., 2017). Paul et al. (2018) reported that land use patterns influenced the soil microbial community structure, mainly by changing soil properties. Sun et al. (2016) also found that soil parent material determined soil bacterial community structure, where the variation of soil properties was the main reason. That may be because the larger variation in soil properties masks the role of land use pattern, soil parent material, and sampling location. This phenomenon should be particularly evident in acidic soils, because soil characteristics including low pH, Al toxicity, P, and N deficiency are more sensitive to soil conditional changes (Guo et al., 2010; Paul et al., 2018). For example, Al toxicity as an important limiting factor in acidic soil has been reported to severely decrease soil microbial abundance and affect microbial diversity (Kunito et al., 2016). The level of Al toxicity is easily changed in acidic soils, because minor pH variation will dramatically alter the concentration of soluble and exchangeable Al. Furthermore, as an important nutrient element, soil P deficiency clearly limits soil microbial community population (Malik et al., 2012), so that slight variations in soil P concentration could significantly affect the *phoD* gene community structure (Tan et al., 2013). Therefore, soil property that is sensitive to anthropogenic disturbances such as fertilization or cropping systems should be the main factor affecting *phoC*-harboring bacterial community in acidic soils.

In a series of soil variables, NO${}_{3}^{-}$ -N, NH${}_{4}^{+}$ -N, C/N ratio, and pH were found to exert significant effects on the *phoC*-harboring bacterial community structure, in which NO${}_{3}^{-}$ -N was the dominant factor (Figure 7 and Supplementary Table 4). Most of the abundant genera correlated closely with these soil variables, especially NO${}_{3}^{-}$ -N (Supplementary Figure 5). Similarly, Wei et al. (2016) found that NO${}_{3}^{-}$ -N significantly affected the PSM community structure during organic waste composting, and pointed out that the growth of PSM was positively influenced by the relatively higher concentration of NO${}_{3}^{-}$ -N. However, Nahas (2007) reported that NH${}_{4}^{+}$ -N was more effective in promoting the growth of PSM than NO${}_{3}^{-}$ -N or organic N sources. It is likely that PSM are taxonomically diverse and respond differently to N sources. The influences of soil N availability and form on *phoC*-harboring microbial community structure should further affect the mineralization of organic P in acidic soils. Additionally, N input tends to accelerate the P cycling rate across a wide variety of habitats, which can be attributed to the increase in phosphatase activity (Marklein and Houlton, 2012). Thus, N sources will lead to enhanced P conservation (Vitousek et al., 2010). On the other hand, relatively low function of nitrification and denitrification processes in acidic soil limit the transformation of the N form (He et al., 2007; Herold et al., 2012), which ultimately may impact the *phoC*-harboring bacterial community structure. These results imply the tight coupling between N and P in acidic soils. Moreover, soil pH has been considered as an important determinant of bacterial community structure in various ecosystems (Geisseler and Scow, 2014), and was also reported as the main driver of *phoD*-harboring community (Ragot et al., 2015). In the current study, soil pH still played an important role in affecting the *phoC*-harboring bacterial community, despite its smaller effect compared to soil NO${}_{3}^{-}$ -N and NH${}_{4}^{+}$ -N (Supplementary Table 4). The effect of soil pH is likely due to the narrow pH ranges for optimal growth of bacteria (Lauber et al., 2009). This result suggested that soil nutrients, especially N-related variables, exert greater effects than soil pH on the soil *phoC*-harboring bacterial community structure in the acidic soil region of southern China.

Although P fertilization has been reported to either increase or decrease the diversity of *phoD*-harboring bacterial community (Chhabra et al., 2013; Tan et al., 2013), soil TP and AP were not found to significantly affect the *phoC*-harboring bacterial community structure in the present study. In acidic soil, low P level may meet the growth of *phoC*-harboring bacteria due to its function, because PSM can mobilize enough P to cover their own needs (Richardson et al., 2009). Unfortunately, although a large number of soil properties, which had been reported as the main variables affecting the community structure and function of microorganism in many studies (Ragot et al., 2015, 2017; Luo et al., 2017), were measured, the explanation rate of these factors is relatively low. Some unmeasured soil properties and biological factors may be attributed to variation of *phoC*-harboring bacterial community. Another plausible explanation is other biotic interactions such as competition and trophic interactions (Stegen et al., 2013). For example, nematode predation promotes *phoD*-harboring bacterial community dynamics in acidic red soil (Jiang et al., 2017), and earthworms in interaction with soil microorganisms increase phosphatase activity (Hoeffner et al., 2019).

## Conclusion

Generally, soil P is an important growth-limiting nutrient for plants and microorganisms in this acidic soil region. The present study demonstrated that *phoC*-harboring bacteria potentially play an important role in improving soil P availability through secreting ACP. Soil TC and TN should contribute to the increase in ACP activity and *phoC* gene abundance, while N-related soil variables including NO${}_{3}^{-}$ -N, NH${}_{4}^{+}$ -N, and C/N mainly determined the *phoC*-harboring bacterial community structure, suggesting the important effect of soil nutrients for regulating the community function of *phoC*-harboring bacteria. This study provides the first investigation of the *phoC*-harboring bacterial community in acidic soil. Future studies will focus on how to effectively stimulate the community function of *phoC*-harboring bacteria through manipulating practices in acidic agricultural soil.

## Data Availability Statement

The datasets generated for this study can be found in the NCBI Sequence Read Archive (SRA) database, SRP198946.

## Author Contributions

CW and RS designed the study. MZ and WS carried out the experiments and data analysis. MZ and WL performed the bioinformatics analysis. MZ and CW were involved in of drafting the manuscript. All authors read and approved the final manuscript.

## Conflict of Interest

The authors declare that the research was conducted in the absence of any commercial or financial relationships that could be construed as a potential conflict of interest.

## Footnotes

1 http://vdb3.soil.csdb.cn/

2 http://www.drive5.com/usearch/

3 http://blast.ncbi.nlm.nih.gov/Blast.cgi
