# Supplementary material for: Soil Nutrients Drive Function and Composition of phoC-Harboring Bacterial Community in Acidic Soils of Southern China
Source: Front Microbiol. 2019 Nov 20;10:2654. doi: 10.3389/fmicb.2019.02654 (PMC6879426; doi:10.3389/fmicb.2019.02654)
Supplement: Supplementary file 1 [file Data_Sheet_1.docx]

**Supplementary Table 1** Completed sampling information across 51 sampling sites in the acidic soil region of southern China.

| Code | Location | Soil parent material | Land use  pattern | Longitude (E) | Latitude (N) | Plant samples | Replicates |
| --- | --- | --- | --- | --- | --- | --- | --- |
| 1 | QY | Red clay | Tea garden | 111°52′16″ | 26°45′35″ | *Camellia sinensis* Linn. | 8 |
| 2 | QY | Red clay | Orchard | 111°52′24″ | 26°45′36″ | *Citrus reticulata* Blanco. | 4 |
| 3 | QY | Red clay | Woodland | 111°52′27″ | 26°45′37″ | *Lespedeza davidii* Franch. | 4 |
| 4 | QY | Red clay | Grassland | 111°52′27″ | 26°45′37″ | *Imperata cylindrica* Linn. | 4 |
| 5 | QY | Red clay | Woodland | 111°52′29″ | 26°45′40″ | *Dicranopteris linearis* Burm. | 4 |
| 6 | QY | Red clay | Woodland | 111°52′26″ | 26°45′41″ | / | / |
| 7 | QY | Red clay | Farmland | 111°52′10″ | 26°45′46″ | *Zea mays* Linn. | 4 |
| 8 | QY | Red clay | Farmland | 111°52′10″ | 26°45′46″ | *Zea mays* Linn. | 4 |
| 9 | QY | Red clay | Farmland | 111°52′10″ | 26°45′46″ | *Zea mays* Linn. | 4 |
| 10 | QY | Red clay | Farmland | 111°52′06″ | 26°45′41″ | *Arachis hypogaea* Linn. | 4 |
| 11 | QY | Red clay | Woodland | 111°52′01″ | 26°45′42″ | *Dalbergia hupeana* Hance. | 4 |
| 12 | QY | Red clay | Woodland | 111°52′05″ | 26°45′42″ | *Liquidambar formosana* Hance. | 4 |
| 13 | QY | Red clay | Orchard | 111°52′05″ | 26°45′41″ | *Citrus reticulata* Blanco. | 4 |
| 14 | QY | Red clay | Grassland | 111°52′07″ | 26°45′43″ | *Imperata cylindrica* Linn. | 4 |
| 15 | QY | Red clay | Farmland | 111°53′04″ | 26°45′23″ | *Zea mays* Linn. | 4 |
| 16 | QY | Red clay | Woodland | 111°53′05″ | 26°45′21″ | *Dicranopteris linearis* Burm. | 4 |
| 17 | QY | Red clay | Tea garden | 111°52′43″ | 26°45′33″ | *Camellia oleifera* Abel. | 8 |
| 18 | JH | Red clay | Woodland | 119°37′08″ | 29°01′03″ | *Osmanthus fragrans* Thunb. | 8 |
| 19 | JH | Red sandstone | Tea garden | 119°35′35″ | 29°00′35″ | *Camellia sinensis* Linn. | 4 |
| 20 | JH | Red clay | Woodland | 119°35′41″ | 29°00′28″ | *Phyllostachys heterocycla* Carr. | 4 |
| 21 | JH | Red clay | Woodland | 119°35′37″ | 28°59′56″ | *Osmanthus fragrans* Thunb. | 3 |
| 22 | JH | Red clay | Orchard | 119°35′34″ | 29°00′12″ | *Eriobotrya japonica* Thunb. | 4 |
| 23 | JH | Red clay | Orchard | 119°35′36″ | 29°00′07″ | *Prunus salicina* Lindl. | 4 |
| 24 | JH | Red clay | Orchard | 119°35′34″ | 29°00′12″ | *Amygdalus persica* Linn. | 4 |
| 25 | JH | Red clay | Orchard | 119°28′55″ | 29°01′51″ | *Citrus maxima* Burm. | 4 |
| 26 | JH | Red clay | Woodland | 119°28′57″ | 29°01′48″ | *Dicranopteris pedata* Houtt. | 4 |
| 27 | JH | Red clay | Woodland | 119°29′46″ | 29°01′42″ | *Carya cathayensis* Sarg. | 4 |
| 28 | JH | Red clay | Woodland | 119°29′46″ | 29°01′42″ | / | / |
| 29 | YT | Red sandstone | Farmland | 116°55′02″ | 28°14′18″ | *Arachis hypogaea* Linn. | 4 |
| 30 | YT | Red sandstone | Woodland | 116°55′02″ | 28°14′42″ | *Castanea mollissima* Bl. | 4 |
| 31 | YT | Red sandstone | Orchard | 116°54′45″ | 28°15′18″ | *Citrus reticulata* Blanco. | 4 |
| 32 | YT | Red sandstone | Woodland | 116°54′45″ | 28°15′18″ | *Dicranopteris pedata* Houtt. | 4 |
| 33 | YT | Red sandstone | Grassland | 116°54′01″ | 28°15′43″ | *Pinus massoniana* Lamb. | 4 |
| 34 | YT | Red sandstone | Woodland | 116°54′01″ | 28°15′43″ | *Castanea mollissima* Bl. | 4 |
| 35 | YT | Red sandstone | Farmland | 116°54′01″ | 28°15′43″ | *Manihot esculenta* Crantz | 4 |
| 36 | YT | Red sandstone | Woodland | 116°54′40″ | 28°15′07″ | *Dicranopteris pedata* Houtt. | 4 |
| 37 | YT | Red sandstone | Farmland | 116°54′40″ | 28°15′07″ | *Oryza sativa* Linn. | 4 |
| 38 | YT | Red clay | Woodland | 116°56′08″ | 28°12′24″ | / | / |
| 39 | YT | Red clay | Grassland | 116°56′16″ | 28°12′37″ | *Pinus massoniana* Lamb. | 8 |
| 40 | YT | Red clay | Woodland | 116°56′08″ | 28°12′31″ | *Pinus elliotti* Engelm. | 4 |
| 41 | YT | Red clay | Woodland | 116°55′55″ | 28°12′32″ | *Lespedeza bicolor* Turcz. | 4 |
| 42 | YT | Red clay | Farmland | 116°55′49″ | 28°12′24″ | *Oxalis corniculata* Linn. | 4 |
| 43 | YT | Red clay | Orchard | 116°55′58″ | 28°12′51″ | *Citrus reticulata* Blanco. | 4 |
| 44 | ZQ | Plate shale | Tea garden | 112°32′17″ | 23°10′46″ | *Camellia sinensis* Linn. | 8 |
| 45 | ZQ | Plate shale | Tea garden | 112°32′20″ | 23°10′46″ | *Dicranopteris linearis* Burm. | 4 |
| 46 | ZQ | Plate shale | Woodland | 112°32′06″ | 23°10′57″ | *Phyllostachys heterocycla* Carr. | 4 |
| 47 | ZQ | Plate shale | Woodland | 112°32′06″ | 23°10′57″ | *Dicranopteris linearis* Burm. | 4 |
| 48 | ZQ | Plate shale | Woodland | 112°32′27″ | 23°10′14″ | *Clausena lansium* Lour. | 4 |
| 49 | ZQ | Plate shale | Woodland | 112°32′30″ | 23°10′12″ | / | / |
| 50 | ZQ | Plate shale | Farmland | 112°33′19″ | 23°09′50″ | *Amaranthus tricolor* Linn. | 4 |
| 51 | ZQ | Plate shale | Grassland | 112°33′23″ | 23°09′55″ | *Dicranopteris linearis* Burm. | 4 |
| Total |  |  |  |  |  |  | 207 |

In abbreviations, QY: Qiyang, Hunan Province; JH: Jinhua, Zhejiang Province; YT: Yingtan, Jiangxi Province; ZQ: Zhaoqing, Guangdong Province.

/ indicates no plant sample. Plant samples of four sampling sites were not collected, because plant diversity was so high that the representative species were not identified.

Replicate numbers per plant at a sampling site are shown in the last column.

**Supplementary Table 2** Primers, program, and reaction composition used for qPCR in the present study.

| Target gene | Primer | Sequence (5'-3') | Size (bp) | Reference | Program | Reaction composition |
| --- | --- | --- | --- | --- | --- | --- |
| 16S rRNA | Eub338 | ACTCCTACGGGAGGCAGCAG | 196 | Fierer et al. (2005) | 95°C 30 S, 50°C 30 S,  72°C 30 S, 40 cycles | 1.0 μl DNA, 0.4 μl forward primers, 0.4 μl reverse primers, 5.0 μl SYBR Premix ExTaq, 3.2 μl sterile distill water |
|  | Eub518 | ATTACCGCGGCTGCTGG |  |  |  |  |
| ITS | ITS1f | TCCGTAGGTGAACCTGCGG | 290 | Fierer et al. (2005) | 95°C 30 S, 55°C 30 S,  72°C 30 S, 40 cycles |  |
|  | 5.8s | CGCTGCGTTCTTCATCG |  |  |  |  |
| *phoC* | phoc-A-F1 | CGGCTCCTATCCGTCCGG | 155 | Fraser et al. (2017) | 95°C 10 S, 58°C 30 S,  72°C 30 S, 40 cycles |  |
|  | phoc-A-R1 | CAACATCGCTTTGCCAGTG |  |  |  |  |

**Supplementary Table 3** Summary of usable sequences after quality filtering and operational taxonomic units (OTUs) across 51 soil samples.

| Code | Location | Sequence number | OTU number |
| --- | --- | --- | --- |
| 1 | QY | 85007 | 79 |
| 2 | QY | 58909 | 77 |
| 3 | QY | 48790 | 56 |
| 4 | QY | 46601 | 96 |
| 5 | QY | 15463 | 34 |
| 6 | QY | 38314 | 49 |
| 7 | QY | 65936 | 87 |
| 8 | QY | 54427 | 54 |
| 9 | QY | 60927 | 89 |
| 10 | QY | 49741 | 76 |
| 11 | QY | 87437 | 75 |
| 12 | QY | 61898 | 55 |
| 13 | QY | 66348 | 123 |
| 14 | QY | 54721 | 134 |
| 15 | QY | 51404 | 81 |
| 16 | QY | 39458 | 86 |
| 17 | QY | 31283 | 123 |
| 18 | JH | 38341 | 81 |
| 19 | JH | 45791 | 72 |
| 20 | JH | 52109 | 122 |
| 21 | JH | 66258 | 126 |
| 22 | JH | 57273 | 157 |
| 23 | JH | 65280 | 142 |
| 24 | JH | 114746 | 130 |
| 25 | JH | 119786 | 83 |
| 26 | JH | 72910 | 52 |
| 27 | JH | 77756 | 69 |
| 28 | JH | 96625 | 226 |
| 29 | YT | 65044 | 188 |
| 30 | YT | 80199 | 224 |
| 31 | YT | 106460 | 236 |
| 32 | YT | 73008 | 184 |
| 33 | YT | 125132 | 246 |
| 34 | YT | 98183 | 219 |
| 35 | YT | 89830 | 190 |
| 36 | YT | 198317 | 250 |
| 37 | YT | 174773 | 277 |
| 38 | YT | 195755 | 266 |
| 39 | YT | 221629 | 245 |
| 40 | YT | 171188 | 208 |
| 41 | YT | 184666 | 258 |
| 42 | YT | 175462 | 226 |
| 43 | YT | 194502 | 219 |
| 44 | ZQ | 129953 | 213 |
| 45 | ZQ | 127813 | 243 |
| 46 | ZQ | 197775 | 281 |
| 47 | ZQ | 127330 | 243 |
| 48 | ZQ | 149958 | 221 |
| 49 | ZQ | 131909 | 240 |
| 50 | ZQ | 115222 | 208 |
| 51 | ZQ | 146925 | 233 |
| Average |  | 98129±52984 | 156±76 |

In abbreviations, QY: Qiyang, Hunan Province; JH: Jinhua, Zhejiang Province; YT: Yingtan, Jiangxi Province; ZQ: Zhaoqing, Guangdong Province.

**Supplementary Table 4** The Monte Carlo test of constrained canonical analysis (CCA) based on *phoC*-harboring bacterial community structure and soil variables.

| Variables | r*^2^* | *p* |
| --- | --- | --- |
| pH | 0.162 | 0.016* |
| TC | 0.022 | 0.476 |
| TN | 0.019 | 0.556 |
| C/N | 0.136 | 0.021* |
| NO_3_^-^-N | 0.681 | 0.001** |
| NH_4_^+^-N | 0.686 | 0.015* |
| TP | 0.014 | 0.667 |
| AP | 0.048 | 0.271 |
| TK | 0.016 | 0.610 |
| AK | 0.075 | 0.144 |

** indicates significantly correlations at *p* < 0.01; * indicates significantly correlations at *p* < 0.05

**Supplementary Figure 1.** Plant TN (A), TP (B), TK (C), TN/TP ratio (D), TN/TK ratio (E), and TP/TK ratio (F) of all collected plant samples.


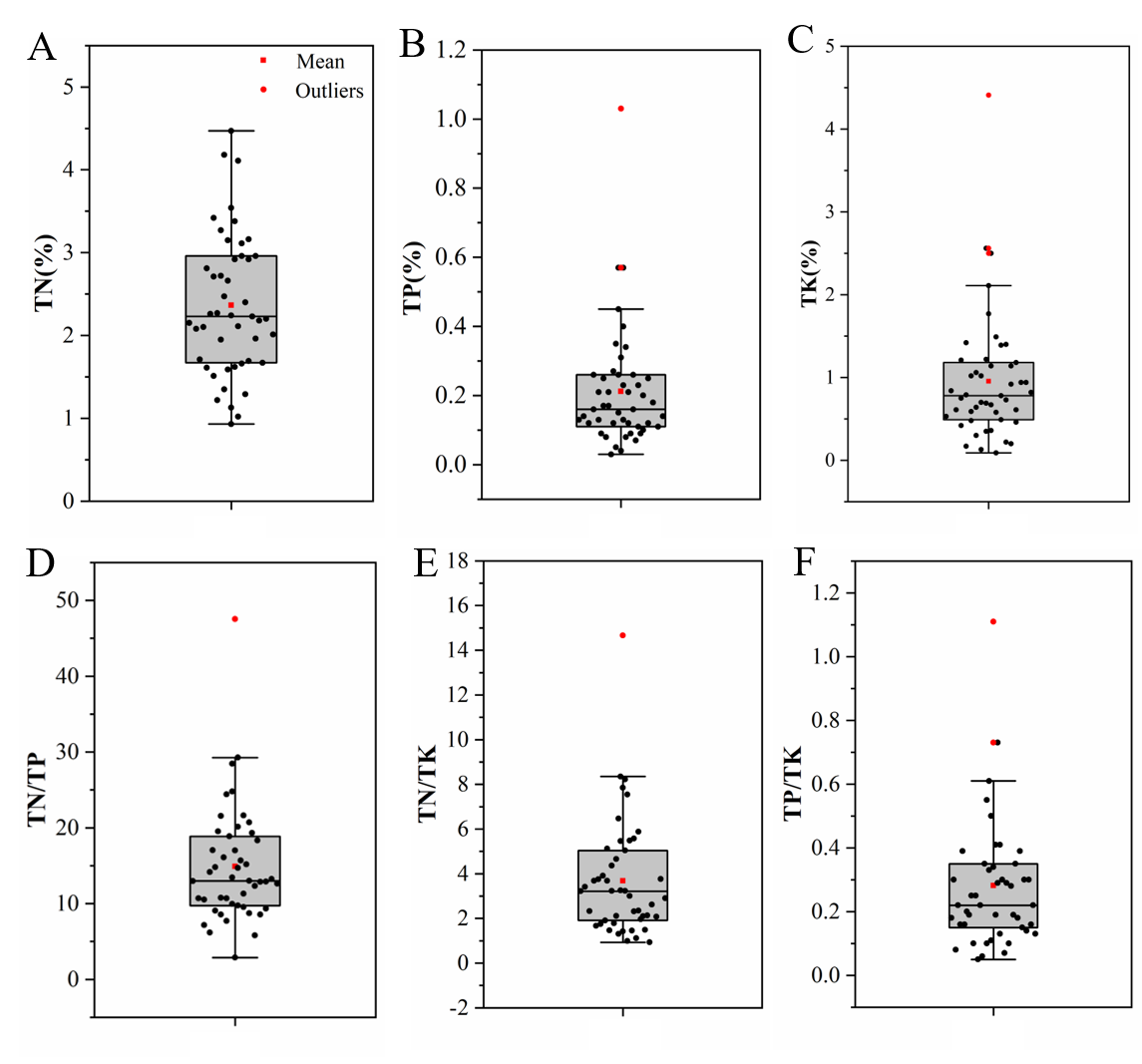


**Supplementary Figure 2.** Soil pH (A), TC (B), TN (C), C/N ratio (D), NO_3_^-^-N (E), NH_4_^+^-N (F), TP (G), AP (H), TK (I), and AK (J) across 51 soil samples.


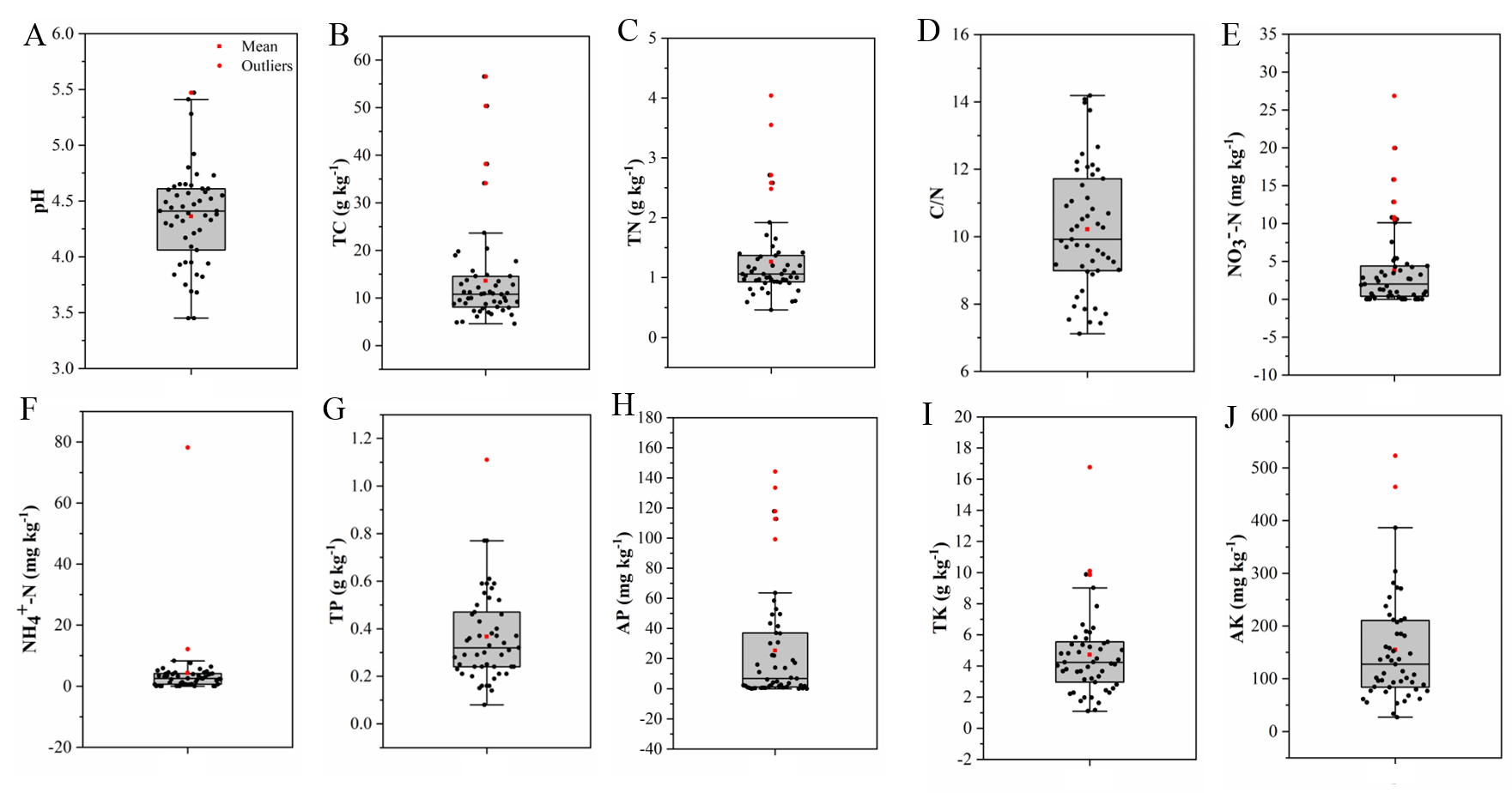


**Supplementary Figure 3.** Gene copy number of 16S rRNA (A), ITS (B), and *phoC* (C) across 51 soil samples.


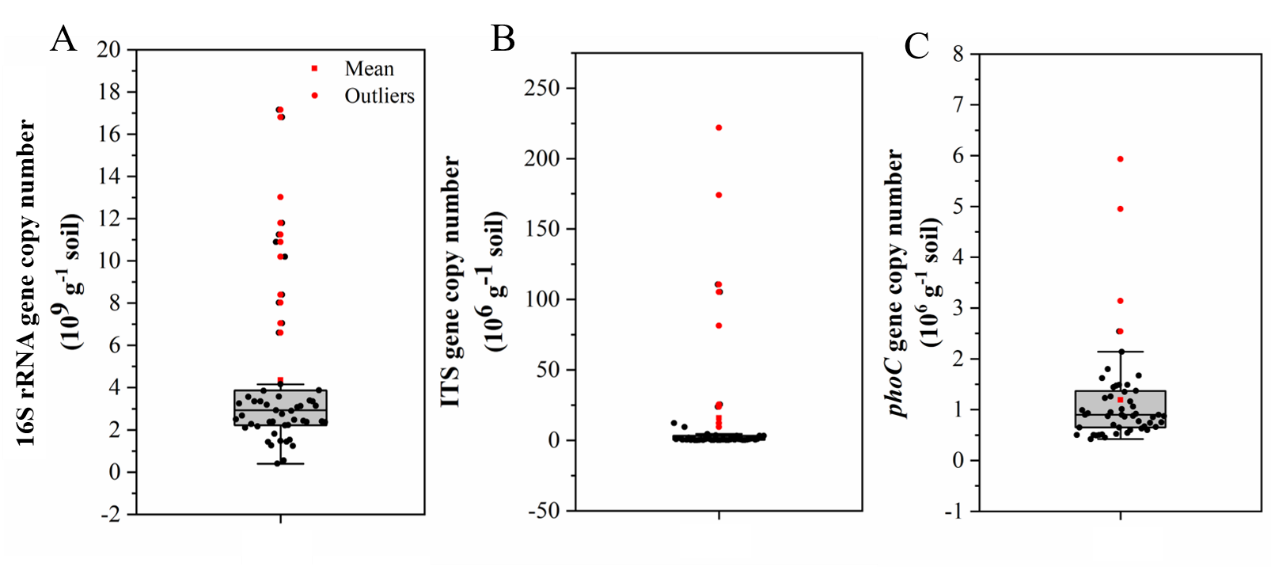


**Supplementary Figure 4.** Spearman’s correlations between OTU number, Shannon index, Simpson index, Chao1 and soil variables across 51 soil samples. ACP indicates ACP activity. * indicates significantly correlations at *p* < 0.05. Blue dots indicate positive correlations, and red dots indicate negative correlations.


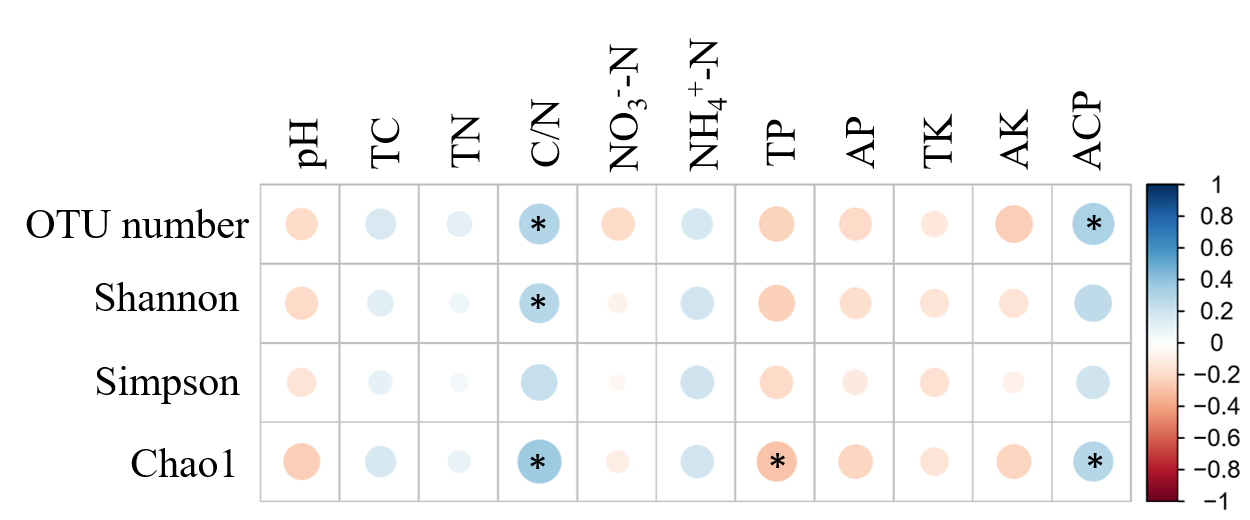


**Supplementary Figure 5.** Spearman’s correlations between the relative abundances of the dominant *phoC*-harboring bacterial genera and soil variables across 51 soil samples. ACP indicates ACP activity. ** indicates significant correlations at *p* < 0.01; * indicates significantly correlations at *p* < 0.05. Blue dots indicate positive correlations, and red dots indicate negative correlations.


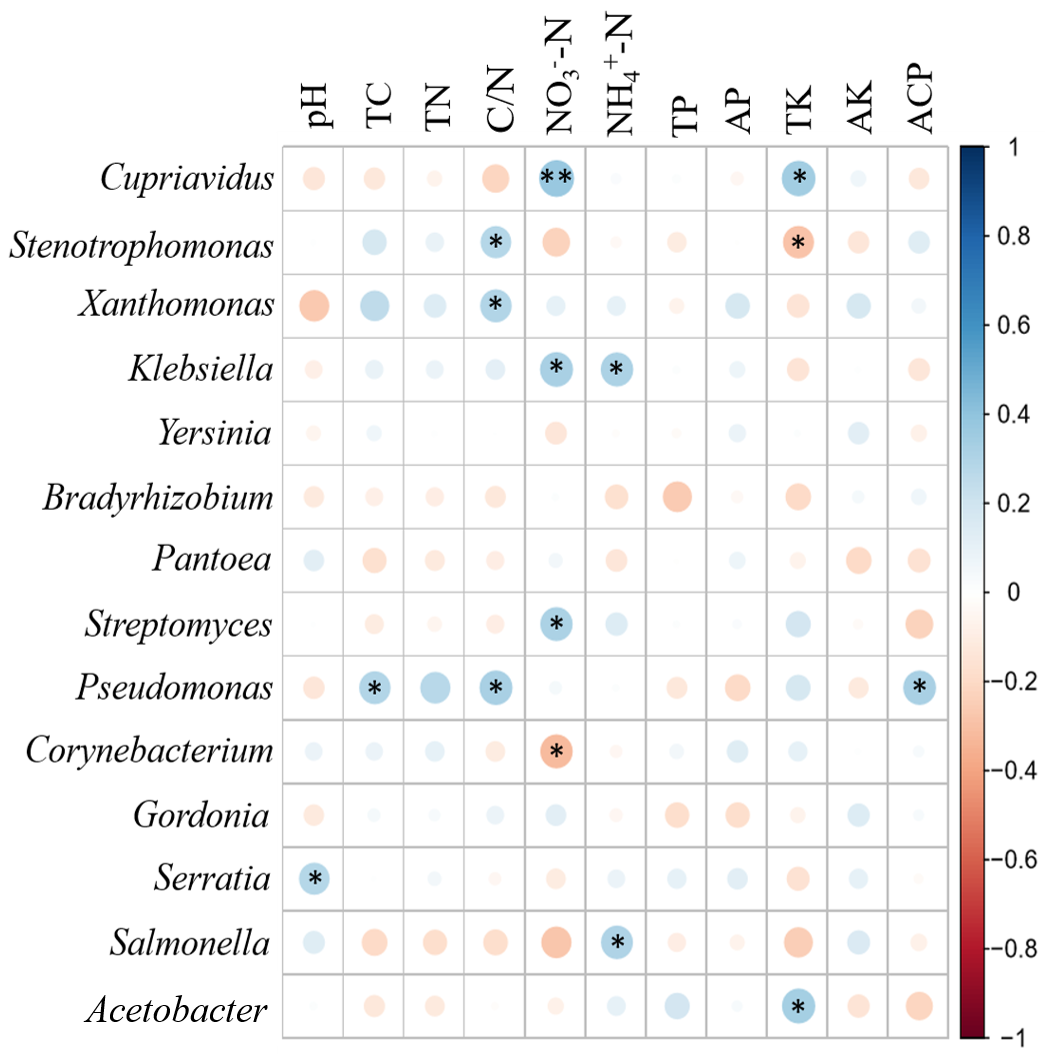


**References**

Fierer, N., Jackson, J.A., Vilgalys, R., and Jackson, R.B. (2005). Assessment of soil microbial community structure by use of Taxon-Specific quantitative PCR assays. *Appl. Environ. Microbiol.* 71, 4117-4120. doi: 10.1128/AEM.71.7.4117-4120.2005

Fraser, T.D., Lynch, D.H., Gaiero, J., Khosla, K., and Dunfield, K.E. (2017). Quantification of bacterial non-specific acid (*phoC*) and alkaline (*phoD*) phosphatase genes in bulk and rhizosphere soil from organically managed soybean fields. *Appl. Soil Ecol.* 111, 48-56. doi: 10.1016/j.apsoil.2016.11.013
